# Structural Variances in Curcumin Degradants: Impact on Obesity in Mice

**DOI:** 10.1021/acs.jafc.4c03768

**Published:** 2024-06-21

**Authors:** Yen-Chun Koh, Han-Wen Hsu, Pin-Yu Ho, Kai-Yu Hsu, Wei-Sheng Lin, Kalyanam Nagabhushanam, Chi-Tang Ho, Min-Hsiung Pan

**Affiliations:** † Institute of Food Sciences and Technology, National Taiwan University, 10617 Taipei, Taiwan; ‡ Department of Food Science, National Quemoy University, 89250 Quemoy County, Taiwan; § Sabinsa Corporation, East Windsor 08520, New Jersey, United States; ∥ Department of Food Science, Rutgers University, New Brunswick 08901, New Jersey, United States; ⊥ Department of Medical Research, China Medical University Hospital, China Medical University, 40402 Taichung City, Taiwan; # Department of Health and Nutrition Biotechnology, Asia University, 41354 Taichung City, Taiwan

**Keywords:** feruloyl acetone, curcumin, degradants, short-chain fatty acid, obesity

## Abstract

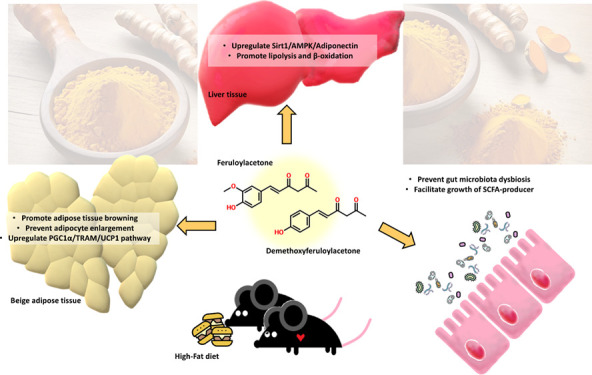

Some thermal degradants of curcuminoids have demonstrated moderate health benefits in previous studies. Feruloyl acetone (FER), recently identified as a thermal degradant of curcumin, has been previously associated with anticancer and antioxidative effects, yet its other capabilities remain unexplored. Moreover, earlier reports suggest that methoxy groups on the aromatic ring may influence the functionality of the curcuminoids. To address these gaps, an animal study was conducted to investigate the antiobesity effects of both FER and its demethoxy counterpart (DFER) on mice subjected to a high-fat diet. The results demonstrated the significant prevention of weight gain and enlargement of the liver and various adipose tissues by both samples. Furthermore, these supplements exhibited a lipid regulatory effect in the liver through the adiponectin/AMPK/SIRT1 pathway, promoted thermogenesis via AMPK/PGC-1α activation, and positively influenced gut-microbial-produced short-chain fatty acid (SCFA) levels. Notably, DFER demonstrated superior overall efficacy in combating obesity, while FER displayed a significant effect in modulating inflammatory responses. It is considered that SCFA may be responsible for the distinct effects of FER and DFER in the animal study. Future studies are anticipated to delve into the efficacy of curcuminoid degradants, encompassing toxicity and pharmacokinetic evaluations.

## Introduction

Turmeric (*Curcuma longa* L.) is a well-known spice in curry powder, renowned for its bioactive orange-yellow compound, curcumin.^1^ Curcuminoids, which include curcumin, demethoxycurcumin (DMC), and bisdemethoxycurcumin (BDMC), constitute the major polyphenols (approximately 2–9%) in turmeric.^2^ Despite the health-beneficial properties highlighted in curcuminoids, efforts are underway to address their limitations regarding efficacy and stability.^3^ Temperature, duration of heating, and changes in pH are major factors leading to the destruction of bioactive compounds in spices.^4^ Previous studies have suggested that curcuminoids are stable against heat (80 °C for 2 h), but they become more susceptible under both acidic and alkaline conditions. The diketone moiety, methoxy groups, and hydroxyl groups may contribute to the distinct degradation behavior of curcuminoids.^5^

India, a developing country, exhibits lower rates of colorectal,^6,7^ prostate,^8^ and lung cancers^9^ compared to other countries. However, the rates of cancer and other chronic diseases have dramatically increased in the offspring generation of Indian immigrants in Western societies, possibly due to distinct dietary patterns.^7^ Although scientific evidence is limited, it is hypothesized that the consumption of dietary turmeric, along with other lifestyle and genetic factors, may contribute to this epidemiologic finding.^10^ Turmeric is typically cooked by boiling or oil-frying, and these processes may lead to the thermal degradation of curcuminoids into several substances, such as deketene curcumin, vanillin, 4-vinyl guaiacol, and ferulic acid.^11^ As an example, it has been demonstrated that roasting curcumin at 180 °C for 70 min produces vanillin, 4-vinyl guaiacol, and ferulic acid as degradation products. Surprisingly, 4-vinyl guaiacol can enhance the transactivation of nuclear factor erythroid 2-related factor 2 (Nrf2) and reduce interleukin-6 gene expression stimulated by lipopolysaccharides.^12^ Additionally, the anticarcinogenic and protective effects of vanillin have been widely studied.^13^ These findings suggest that such thermal degradants might contribute to the health benefits of heated turmeric in India. The identification of these thermal degradants and their potential health benefits could have implications for the traditional use of heated turmeric in India. These implications include dietary suggestions, modifications in the processing procedures of turmeric-containing foods, providing scientific evidence for the medicinal use of turmeric, and stimulating further studies on these thermal degradants.

Deketene curcumin is produced from curcumin via pyrolytic formation, and vanillin and ferulic acid have been identified as two degradation products in this process. It is worth mentioning that, in addition to vanillin, feruloyl acetone (FER) is another intermediate product resulting from the cleavage of the α,β-unsaturated bridge of curcumin.^14^ Another study suggested that the proportion of degraded curcumin transformed into FER, when heated at 150 °C for 2 h under either alkaline or acidic conditions, was up to 28.8 and 20.6%, respectively. It was proposed that FER could be the major transformation product of curcumin.^15^ Therefore, FER could also contribute to the benefits of cooked turmeric.

Among various phytochemicals, curcumin has garnered attention for its significant health benefits and safety for consumption.^16^ As previously mentioned, curcuminoids and turmeric-containing foods could be relevant to the low incidence of certain cancers in India. In addition to its potential anticancer properties, numerous reviews suggest that curcumin can help ameliorate obesity through multiple mechanisms.^17^ These include maintaining redox balance,^18^ reducing inflammation,^19^ modulating gut microbiota,^20^ inhibiting adipogenesis,^21^ stimulating energy expenditure,^22^ and regulating lipid metabolism.^23^ Furthermore, our previous study showed that the curcumin degradant, FER, exhibited an anticancer effect, but its potential antiobesity effect has not been discussed. Additionally, it was revealed that its demethoxylated counterpart exhibited a better effect on antioxidation. Therefore, this study aims to determine the preventive effect of FER and demethoxy FER (DFER) on diet-induced obesity and elucidate the underlying mechanisms.

## Materials and Methods

### Materials

FER and DFER, with purities exceeding 95%, were provided by Sabinsa Corporation (New Jersey, USA). LC-MS chromatograms of curcuminoids, FER, and DFER are presented in Figure S1. Antibodies against anti-p-AMPK and AMPK were obtained from Cell Signaling Technology (MA, USA), while anti-ATGL was purchased from Santa Cruz Biotechnology (Texas, USA). Additionally, antibodies against PPARα, PRDM16, and GAPDH were sourced from Abcam PLC (Cambridge, UK), while antibodies against CPT-1A, PGC-1α, adiponectin, and UCP-1 were acquired from Proteintech (IL, USA).

### In Vitro Study—Lipid Accumulation in Adipocytes

The 3T3-L1 murine preadipocyte cell line was purchased from ATCC and was employed to assess the antiadipogenesis capability in an in vitro study. Cells were cultured in DMEM medium supplemented with 10% FBS and were initially seeded at a density of 2 × 10^4^ cells/mL and allowed to grow for 3 days until reaching confluence. The medium was then changed from 10% FCS-supplemented DMEM to 10% FBS-supplemented DMEM, and cells were allowed to adapt for 2 days. Subsequently, the medium was replaced with differentiation medium I on day 0, supplemented with insulin, dexamethasone, rosiglitazone, and 3-isobutyl-1-methylxanthine, and differentiation was allowed to proceed for 2 days. Following this, the medium was switched to differentiation medium II on day 2, supplemented with insulin, for an additional 2 days. The medium was changed every 2 days, and samples were provided (at various concentrations) whenever the medium was replaced. On day 8, the cells were fixed with 4% formaldehyde and stained with oil red O solution. After images were captured, oil red O was dissolved using isopropanol, and the absorbance was measured at a wavelength of 500 nm.

### In Vitro Study—Lipid Accumulation in Hepatocytes

The HepG2 cell line was purchased from ATCC and was employed to assess the preventive effect of the sample on lipid accumulation. The cells were cultured in DMEM medium supplemented with 10% FBS, initially seeded at a density of 4 × 10^5^ cells/mL, and allowed to grow for 24 h. BSA-conjugated oleic acid (OA) was added into the medium at a concentration of 500 μM, with or without the samples. After 24 h treatment, the cells were fixed with 4% formaldehyde and stained with oil red O solution. After capturing images, oil red O was dissolved using isopropanol, and the absorbance was measured at a wavelength of 500 nm.

### In Vitro Study—Nitrite Production in Macrophages

The murine macrophage RAW264.7 cell line was purchased from ATCC and was employed to assess the anti-inflammatory effect of the samples. The cells were cultured in DMEM medium supplemented with 10% FBS and were initially seeded at a density of 1 × 10^6^ cells/mL. After 12 h, the cells were treated with LPS (100 ng/mL) for 24 h with or without the samples at various concentrations in the DMEM medium without FBS supplementation. The medium was mixed with Griess reagent, and the absorbance was measure at a wavelength of 550 nm.

### Animal Study Design

A total of 40 male C57BL/6 mice were obtained from the National Laboratory Animal Center (Taipei, Taiwan). The mice were housed under controlled environmental conditions at a temperature of 25 ± 1 °C and a relative humidity of 50%. The animal study was conducted following ethical guidelines and received approval from the Institutional Animal Care and Use Committee (IACUC) of the National Taiwan University, with approval number NTU-111-EL-00088. After an adaptation period of 1 week, the mice were divided into four groups, each consisting of 10 mice. The control group was provided with a standard chow diet (Purina 5001, Lab Diet), while the remaining groups received a high-fat diet (HFD), which included 50% of the calories derived from fat. The diet was supplemented with FER and DFER at a dosage of 0.25% (w/w) for 16 weeks. Body weight measurements were taken weekly, and at the end of the 16-week study period, the mice were humanely euthanized using CO_2_ asphyxiation. Subsequently, their organs were weighed, photographed, and preserved at −80 °C for further analysis.

### Hematoxylin and Eosin (H&E) Staining Procedure

For histopathological examination, the liver and three distinct adipose tissues underwent H&E staining to facilitate the visualization of structural nuances. Initially, collected tissue samples were fixed in 10% formalin buffer solution. Following fixation, tissues underwent dehydration and subsequent paraffin embedding. The paraffin-embedded tissues were then sectioned into thin slices, measuring 3–5 μm in thickness. Subsequently, these tissue sections underwent deparaffinization, rehydration, and the H&E staining protocol. The assessment included examining the presence of empty vacuoles and evaluating adipocyte sizes. The size distribution of adipose tissue adipocytes was determined using ImageJ software.

### Western Blotting Procedure

Liver and fat tissues were homogenized and subsequently lysed with an ice-cold lysis buffer, allowing a minimum 1 h incubation time on ice. After homogenization, samples underwent centrifugation at 14,000*g* for 1 h at 4 °C. The resulting supernatants were carefully collected and stored at −80 °C until further use. Protein lysate concentrations were determined by using a Bio-Rad protein assay. For electrophoresis, 25 μg of protein samples was loaded into individual wells and transferred onto PVDF membranes (Merck Millipore Ltd., Tullagreen, County Cork, Ireland). Following transfer, membranes underwent a blocking procedure with blocking solution, followed by overnight incubation with primary antibodies at 4 °C. To ensure adequate antibody binding and removal of unbound antibodies, membranes underwent 10 min washes with a solution consisting of 0.2% phosphate-buffered saline Tween 20 (TPBS) for three times, both before and after the application of secondary antibodies. Protein band visualization was achieved through chemiluminescence (ECL, Merck Millipore Ltd.), and densitometry analysis of the bands was performed using ImageJ imaging software. GAPDH served as an internal control for Western blotting.

### Short-Chain Fatty Acids Analysis

3–4 steel beads were placed in a fecal homogenization tube. 0.1 g of colonic feces was mixed with 0.5 mL of 0.5% phosphoric acid solution and homogenized at 7 m/s for 15 s (2 cycles). The sample was centrifuged to reduce foam; then 0.75 mL of ethyl acetate was added, and the sample was homogenized again at the same settings. After centrifuging at 18,000 rpm for 10 min at 4 °C, 0.2 mL of the supernatant was mixed with 0.8 mL of 625 μM 4-methylvaleric acid internal standard. This mixture was filtered using a 0.22 μm nylon syringe filter in a vial for analysis. Analysis was performed using a DB-WAXetr column with a 1 mL/min flow rate in the splitless mode. The column temperature was 250 °C, auxiliary gas temperature 280 °C, ion source temperature 230 °C, and quadrupole temperature 150 °C. The *m*/*z* detection range was 30–250, with a solvent delay time of 3.5 min. The oven temperature program started at 90 °C, increased to 150 °C at 15 °C/min over 4 min, to 170 °C at 5 °C/min over the next 4 min, and to 250 °C at 20 °C/min over the following 4 min, holding at 250 °C for the remaining 2 min.

### Gut Microbiota Analysis

Colonic feces were collected and stored at–80 °C. Fecal microbial DNA extraction and purification were performed using the innuPREP Stool DNA isolation kit. Designed primers containing a 5′ buffer sequence (GCATC) with a 5′ phosphate modification were utilized for genomic DNA amplification. HiFi reads with a predicted accuracy (Phred Scale) of 30 were generated using the PacBio Sequel IIe instrument in the circular consensus sequence (CCS) mode.

### Statistical Analysis

The results were presented as mean ± standard error of the mean (S.E.M), and the significant differences between each group were determined by the student’s *t* test. A *p*-value <0.05 was considered statistically significant in this study.

## Results

### DFER Showed a Greater Effect on Preventing Weight Gain in HFD-Induced Obesity in Mice

**Figure 1:**
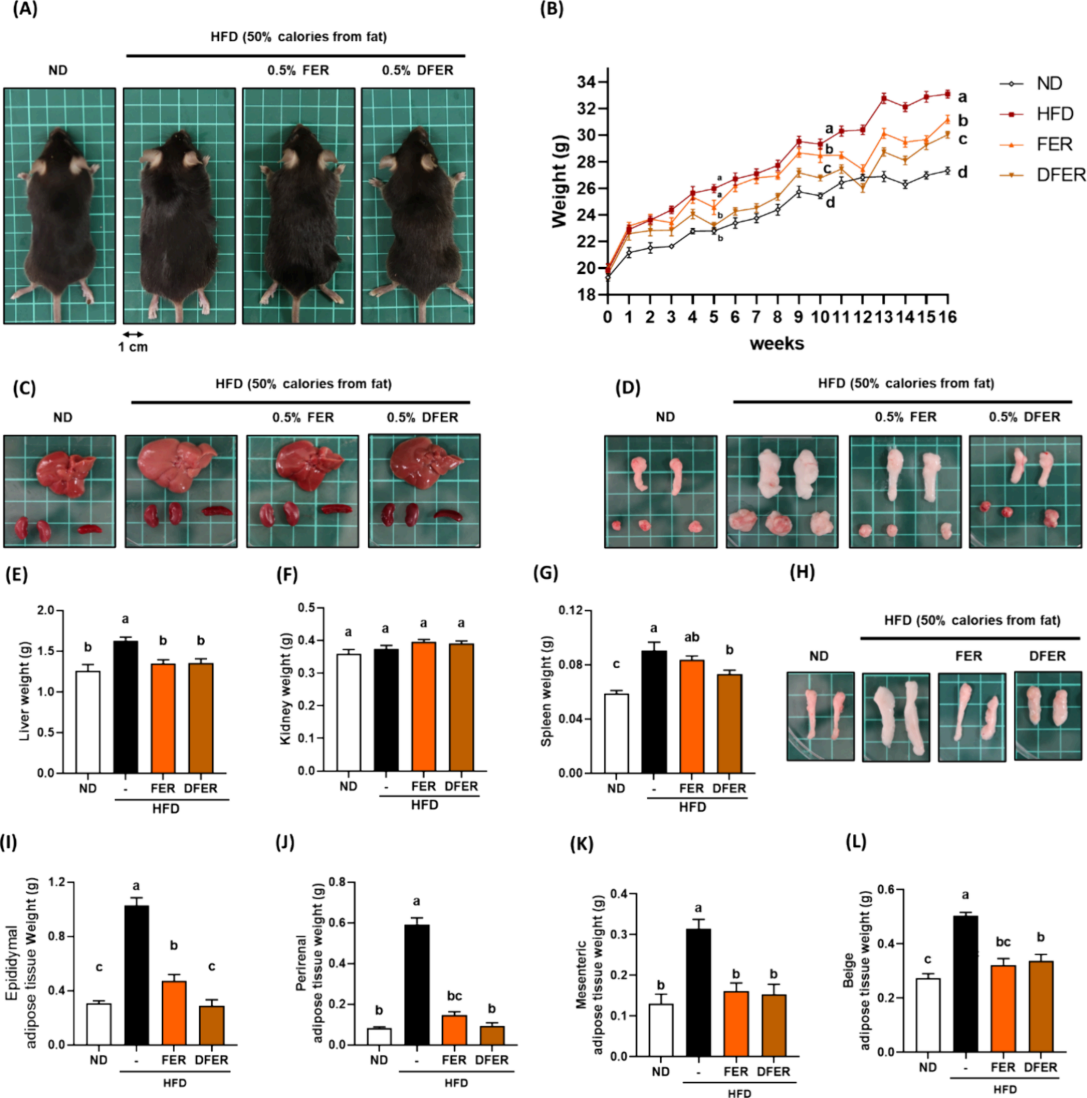
DFER showed a greater effect on preventing weight gain in HFD-induced obesity in mice. (A) Representative appearance of mice from each group, (B) body weight change over weeks, (C) organs (liver, kidney, and spleen), (D) adipose tissues (epididymal, perirenal, and mesenteric adipose tissues), the average weight of (E) liver, (F) kidney, and (G) spleen, (H) representative appearance of beige adipose tissue, and the average weight of (I) epididymal WAT, (J) perirenal WAT, (K) mesenteric WAT, and (L) beige adipose tissue. All the data are presented as mean ± S.E, *n* = 10. Different lowercase letters indicate a significant difference among the groups, as determined by ANOVA followed by Duncan’s post hoc test.

The representative appearances and body weight changes over weeks are presented in Figure 1A,B, respectively. The body weight of mice fed a high-fat diet (HFD) significantly increased, and a significant reduction was observed in the DFER group at week 5 but not in the FER group. By week 10, both sample groups exhibited an observable effect on body weight reduction compared with the HFD group. It is noteworthy that the DFER group had a greater impact than the FER group in terms of body weight reduction (Figure 1B). These significant differences persisted until the end of week 16, highlighting the compelling effect of DFER in preventing body weight gain. The changes in body weight were also reflected in organ and tissue weights.

The average final weight of the HFD group reached 33.09 ± 0.29 g, whereas the average body weights of the supplemented groups were significantly lower (Table 1). However, there was no obvious difference in the daily food intake and daily water intake among the groups fed with the HFD, suggesting that the reduction in body weight was not attributable to a decrease in food or water intake.

**Table 1:** Body Weight Index, Food Intake, Water Intake, and Serum Biochemical Parameters^a^

	ND	HFD		
			0.5% FER	0.5% DFER
initial weight (g)	19.30 ± 0.27^a^	19.83 ± 0.24^a^	20.00 ± 0.29^a^	19.55 ± 0.38^a^
final weight (g)	27.27 ± 0.29^d^	33.09 ± 0.29^a^	31.19 ± 0.31^b^	30.03 ± 0.25^c^
weight gain (g)	8.03 ± 0.32^c^	13.26 ± 0.39^a^	11.19 ± 0.48^b^	10.51 ± 0.43^b^
food intake (g/day)	4.10 ± 0.10^a^	3.19 ± 0.07^b^	3.13 ± 0.10^b^	3.06 ± 0.10^b^
water intake (mL/day)	5.08 ± 0.23^a^	3.97 ± 0.17^b^	3.92 ± 0.20^b^	4.10 ± 0.21^b^
serum biochemical parameters				
AST (mg/dL)	134.95 ± 2.20^bc^	180.47 ± 15.77^a^	87.75 ± 3.86^c^	159.8 ± 1.60^bc^
ALT (mg/dL)	36.18 ± 0.46^b^	63.74 ± 9.71^a^	29.38 ± 3.50^b^	40.72 ± 5.70^b^
TG (mg/dL)	74.92 ± 10.04^a^	70.84 ± 6.28^a^	40.66 ± 7.47^b^	36.28 ± 1.08^b^
T-CHO (mg/dL)	88.31 ± 4.80^c^	194.98 ± 8.57^a^	131.26 ± 7.57^b^	134.18 ± 11.84^b^
HDL (mg/dL)	78.00 ± 2.20^b^	119.68 ± 15.77^a^	88.14 ± 3.86^b^	92.16 ± 1.60^b^
LDL (mg/dL)	11.67 ± 0.58^b^	56.15 ± 2.62^a^	44.35 ± 4.71^a^	47.67 ± 8.11^a^
NEFA (Eq/L)	720.88 ± 175.40^b^	904.63 ± 146.99^a^	689.63 ± 150.48^b^	672.13 ± 50.96^b^

a Different lowercase letters indicate a significant difference among the groups, as determined by ANOVA followed by Duncan’s post hoc test. AST, aspartate aminotransferase; ALT, alanine transaminase; TG, triglycerides; T-CHO, total cholesterol; HDL, high-density lipoprotein; LDL, low-density lipoprotein; NEFA, nonesterified fatty acid.

### The Effect of FER and DFER on Organs and Fat Tissues of HFD-Fed Mice

Figure 1C,D depicts the representative appearance of the mice liver, kidneys, and spleen and epididymal white adipose tissues (WATs), perirenal WATs, and mesenteric WATs, respectively. Paler and larger appearances of the liver and WAT tissues were observed, and these observations were also reflected in the weights (Figure 1E,I–K). Both FER and DFER supplementation significantly prevented the enlargement of these tissues, with DFER demonstrating a superior effect, particularly in terms of spleen and epididymal WAT weights (Figure 1G,I). In addition to the typical WATs, brown-like WAT (beige adipose tissue) also contributed to the reduction of the body weight in both sample groups (Figure 1H,L).

### Both FER and DFER Supplementation Prevent Lipid Accumulation in the Liver and WATs of HFD-Fed Mice

**Figure 2:**
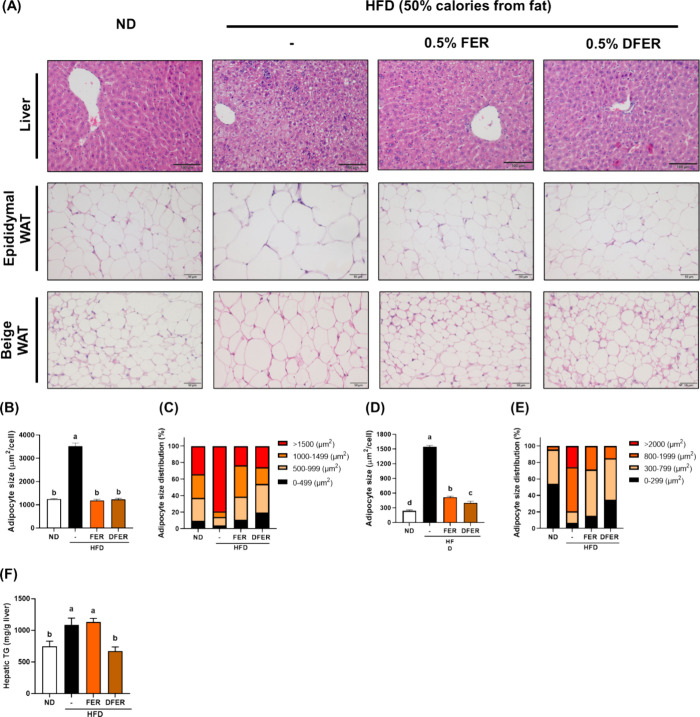
Both FER and DFER supplementation prevent lipid accumulation in the liver and white adipose tissues (WATs) of HFD-fed mice. (A) H&E staining of liver, epididymal WAT, and beige WAT sections. The liver sections were viewed at 200× magnification (scale bar = 100 μm), while the WATs were viewed at 400× magnification (scale bar = 50 μm) under a light microscope. (B) Average adipocyte size and (C) adipocyte size distribution (%) of epididymal adipose tissue. (D) Average adipocyte size and (E) adipocyte size distribution (%) of beige adipose tissue. (F) Hepatic TG content. Different lowercase letters indicate a significant difference among the groups, as determined by ANOVA followed by Duncan’s post hoc test.

To further confirm the beneficial effects of FER and DFER on lipid metabolism, H&E staining was conducted. As shown in Figure 2A, observable lipid vacuoles, including micro- and macrovesicular types, were found in the liver section of the HFD group, along with immune cell infiltration. In comparison, both samples markedly prevented severe lipid accumulation and macrophage infiltration. Small lipid vacuoles were still observed in the FER group, suggesting a better preventive effect of DFER, but further investigation is needed before a conclusion can be drawn.

**Figure 3:**
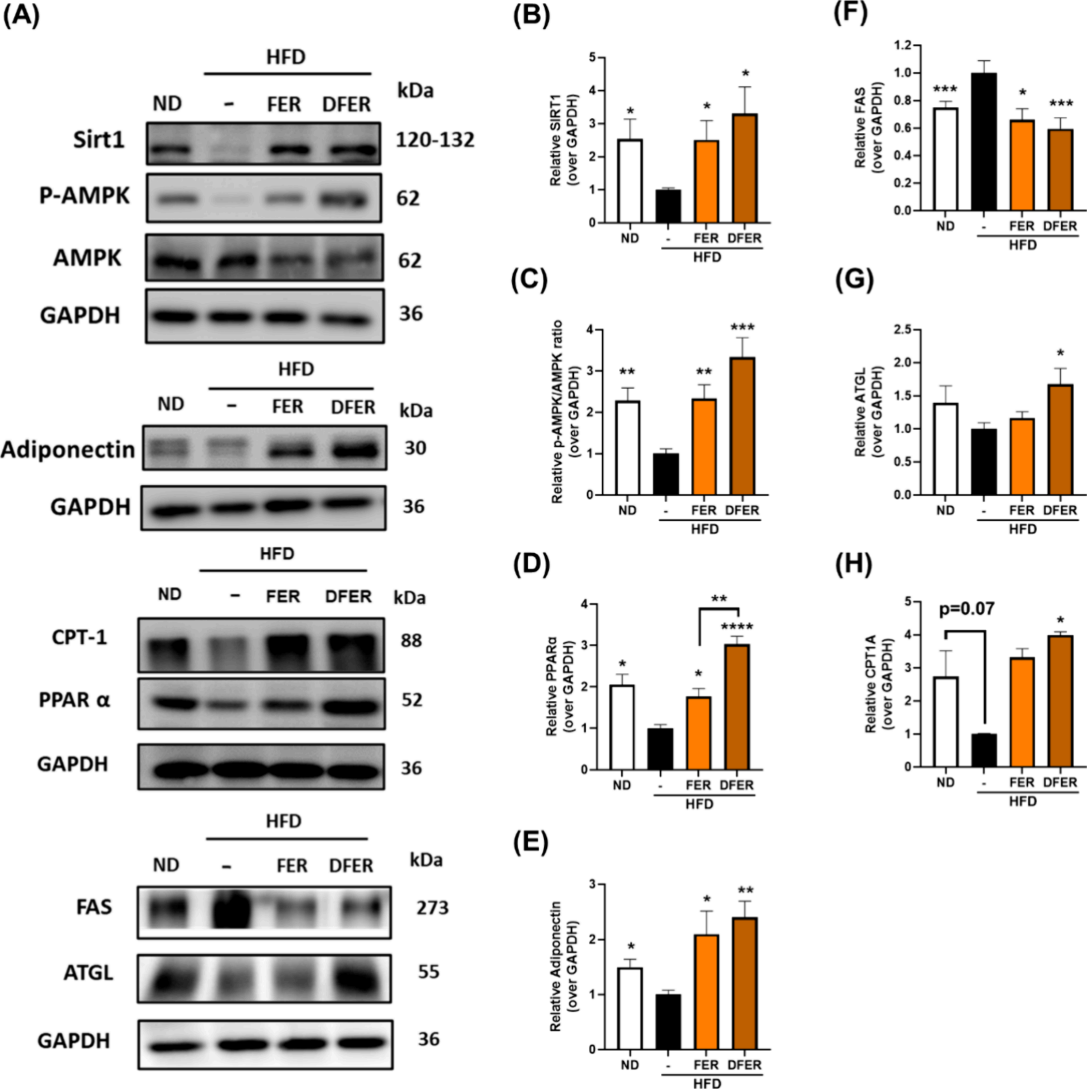
Dietary supplementation with DFER promotes the hepatic adiponectin/AMPK/Sirt1 pathway to prevent lipid accumulation. (A) Representative images of the proteins using Western blot and the relative quantification of hepatic proteins: (B) SIRT1, (C) p-AMPK/AMPK ratio, (D) PPARα, (E) adiponectin, (F) FAS, (G) ATGL, and (H) CPT1A, with GAPDH used as an internal control. The symbols (*), (**), (***), and (****) indicate a significant difference when compared to the HFD group, with *p*-values less than 0.05, 0.01, 0.005, and 0.001, respectively.

The results of H&E stains of epididymal and beige WATs indicated that both samples were effective in preventing adipocyte enlargement. After conducting adipose size quantification with ImageJ software, it was confirmed that both samples significantly anticipated adipocyte enlargement (Figure 2B–E). Although there was no distinct difference in average adipocyte size between the FER and DFER groups (Figure 3B), the adipocyte size distribution (Figure 2C) showed that DFER consisted of a large number of smaller adipocytes (<500 μm^2^). Moreover, the average adipocyte size of beige adipose tissue in DFER (Figure 2D) was significantly lower than that in the FER group. These findings imply that DFER could have a more notable effect on lipid metabolism compared to that of FER.

### The Effect of FER and DFER on Serum Biochemical Parameters

The serum biochemical parameters of each group are presented in Table 1. Both AST and ALT parameters are significantly increased in the HFD groups, suggesting a certain degree of hepatic damage caused by lipid accumulation. Both samples could prevent the elevation of AST and ALT in the serum, implying a hepatic protective effect of these samples against HFD feeding. Regarding the serum lipid profiles, both samples effectively reduced TG, T-CHO, HDL, and NEFA, but the supplementations could not reverse the elevation of serum LDL induced by HFD feeding. However, there were no significant differences in these parameters between the FER and DFER groups. Therefore, the underlying mechanism to promote lipid metabolism was determined in the following section.

### Dietary Supplementation of DFER Promotes the Hepatic Adiponectin/AMPK/SIRT1 Pathway to Prevent Lipid Accumulation

To investigate the underlying mechanism preventing hepatic lipid accumulation, a Western blot analysis was conducted. Activation of AMPK was observed in both sample groups (Figure 3A,C), accompanied by an upregulation of SIRT1 expression (Figure 3A,B). Additionally, the adiponectin level was significantly increased in both supplemented groups (Figure 3A,E). The elevation of both adiponectin and SIRT1 might potentially induce the activation of AMPK to promote lipid metabolism. To confirm the role of activated AMPK in lipid metabolism, downstream proteins were investigated. The reduction in FAS expression and increased ATGL level suggested that AMPK activation could lead to decreased lipogenesis in both sample groups (Figure 3A,F). However, ATGL expression was induced only in the DFER group, suggesting that the induction of hepatic lipolysis was greater in this group. This result was further evidenced by the degree of hepatic β-oxidation, where the higher protein levels of PPARα and CPT1A in the DFER group indicated that the fatty acids produced from lipolysis would be consumed, explaining the promoted β-oxidation in the DFER group (Figure 3A,D,H). The result was also supported by the H&E staining, which presented some microvesiculars in the FER group (Figure 2A).

### DFER Induces AMPK/PGC-1α to Promote Browning in Inguinal WAT

**Figure 4:**
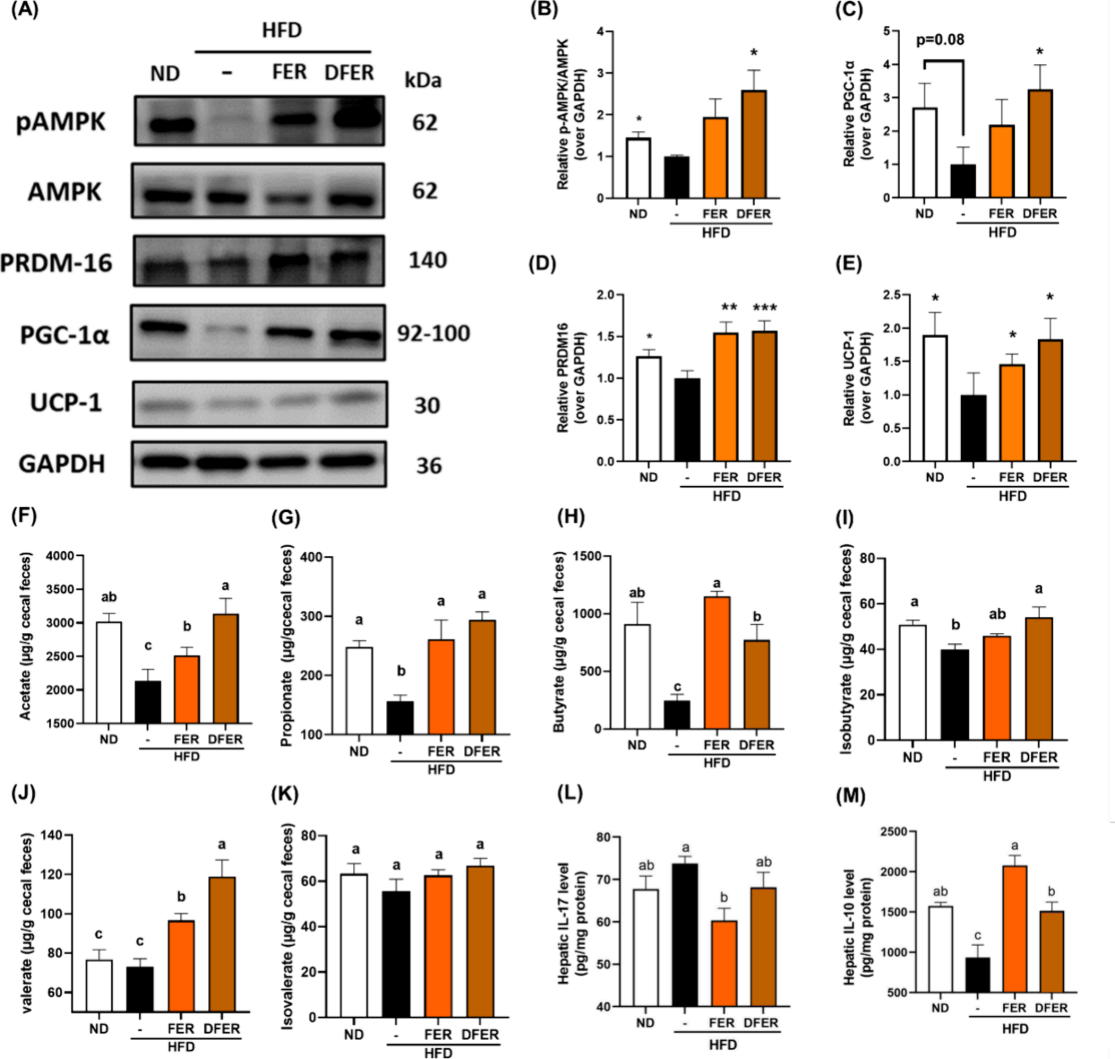
DFER induces AMPK/PGC-1α to promote browning in inguinal WAT. (A) Representative images of the proteins using Western blot and the relative quantification of hepatic proteins: (B) p-AMPK/AMPK ratio, (C) PGC-1α, (D) PRDM16, and (E) UCP-1, with GAPDH used as an internal control. The cecal (F) acetate, (G) propionate, (H) butyrate, (I) isobutyrate, (J) valeric acid, and (K) isovaleric acid contents. Hepatic (L) IL-17 and (M) IL-10 levels. The symbols (*), (**), and (***) indicate a significant difference compared to the HFD group, with p-values of less than 0.05, 0.01, and 0.005, respectively. Different lowercase letters indicate a significant difference among the groups, as determined by ANOVA followed by Duncan’s post hoc test.

Activation of AMPK was also observed in the inguinal WAT. A significant increase in the p-AMPK/AMPK ratio was found in the DFER group but not in the FER group. Similarly, the downstream protein of AMPK, PGC-1α, was found to elevate only in the DFER group (Figure 4A,B,D). However, the expressions of other thermogenesis-related proteins, PRDM16 and UCP-1, were found to be induced in both sample groups, but at a higher level in the DFER group. The results indicated that both samples could promote thermogenesis in inguinal WAT, but the activation of the AMPK/PGC-1α pathway by DFER, to a large extent, led to a greater degree of fat browning. These findings are also reflected in the average adipocyte size and their distribution (Figure 2D,E).

### Modulatory Effect of FER and DFER on Gut Microbial Short-Chain Fatty Acids (SCFAs)

In our previous study, a positive correlation between SCFA and lipid metabolism was found.^24^ Therefore, various cecal SCFA contents were determined. Surprisingly, FER and DFER showed distinct effects on the SCFA composition. In comparison, dietary FER could lead to an incremental increase in cecal butyric acid and valeric acid levels and reverse the propionic acid reduction caused by HFD feeding (Figure 4E,F,H). Meanwhile, higher levels of acetic, isobutyric, and valeric acids were observed in the DFER group.

**Table 2:**
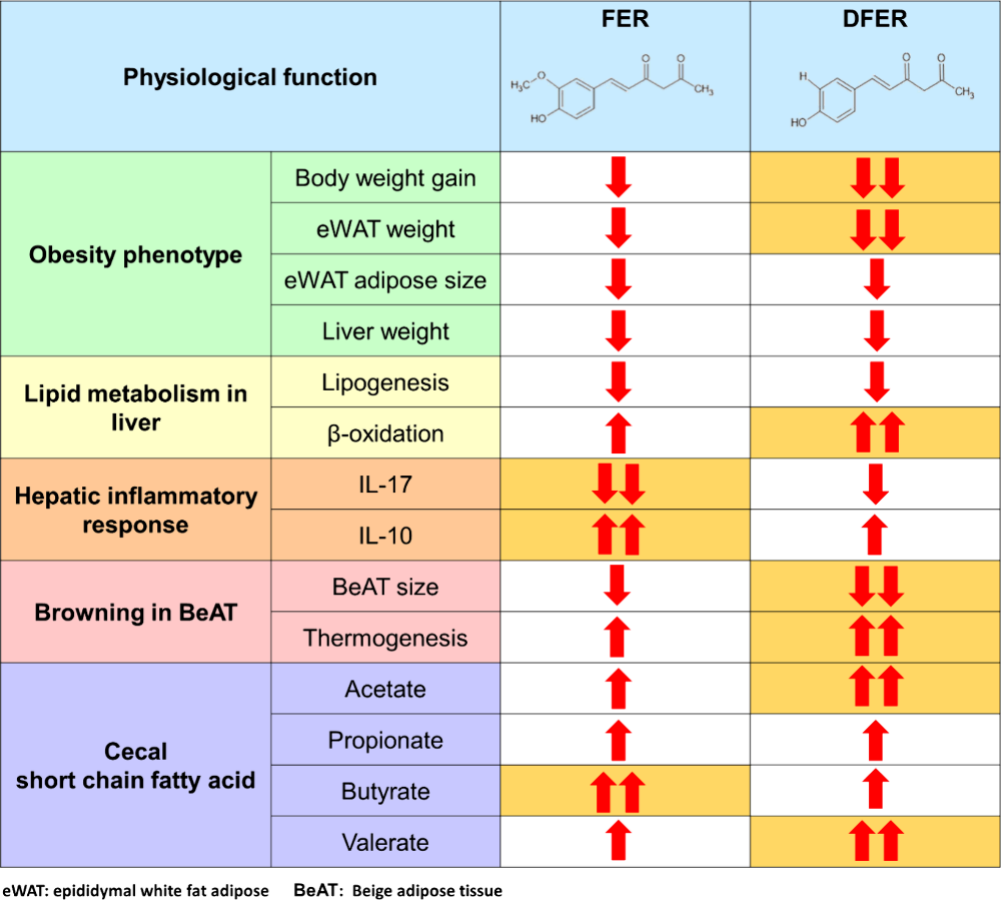
Comparison of the Physiological Function Brought by Dietary Supplementation of FER and DFER^a^

a eWAT: epididymal white fat adipose BeAT: beige adipose tissue.

Notably, the immune cell infiltration was observed in the hepatic H&E staining (Figure 3A). It was previously reported that SCFA such as butyric acid could modulate functional immune cells via IL-10 and IL-17. Our results showed that FER had a greater effect on modulating immune responses than DFER (Figure 4J,K). However, further investigations are needed to confirm our finding that FER and DFER could show distinct health benefits.

**Figure 5:**
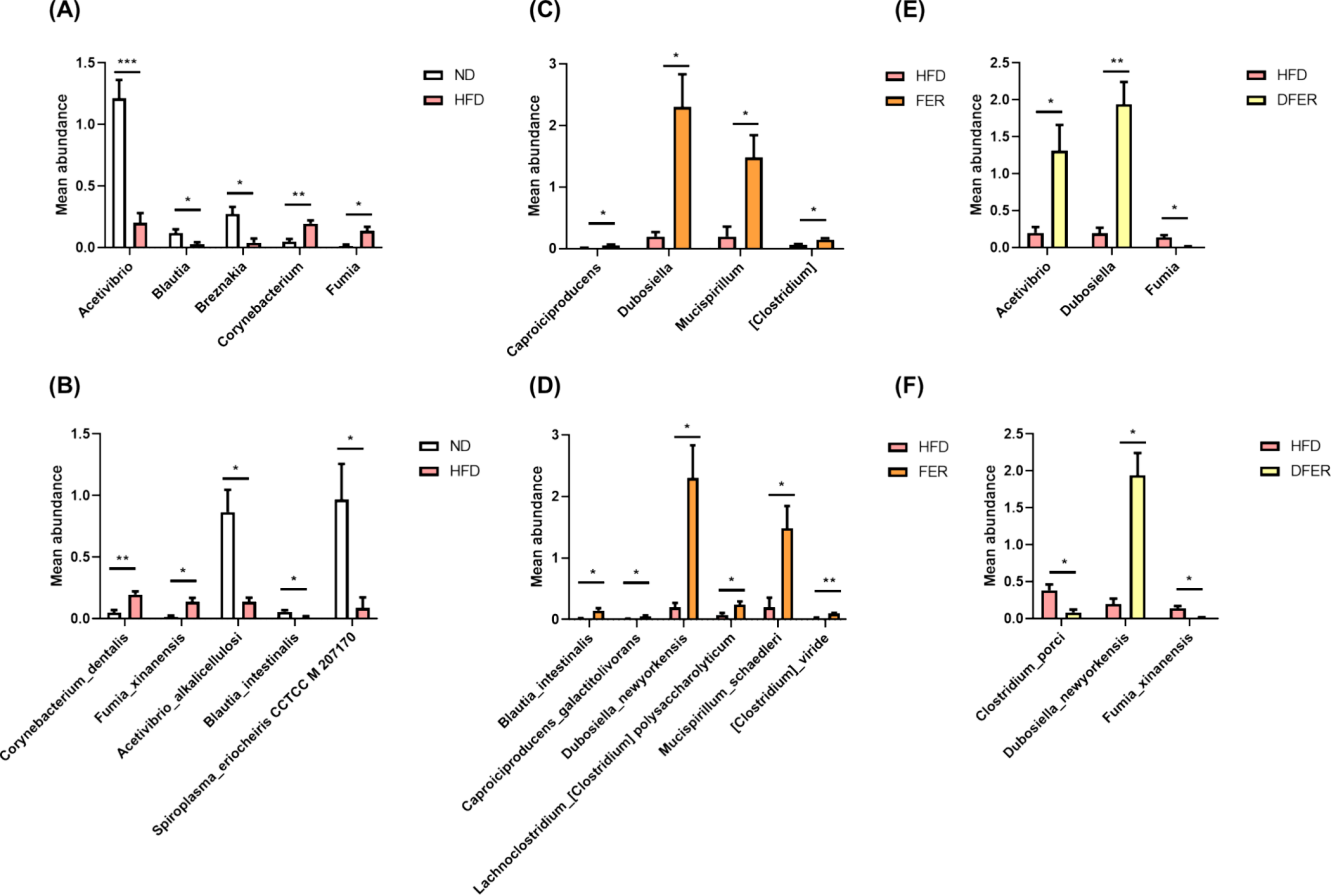
Gut microbial differences between compared groups by using Welch’s *t* test in Statistical Analysis of Metagenomic Profiles (STAMP) software. Mean abundances in taxonomic rank of genus, (A) ND vs HFD, (C) HFD vs FER, (E) HFD vs DFER, and mean abundances in taxonomic rank of species, (B) ND vs HFD, (D) HFD vs FER, and (F) HFD vs DFER. The symbols (*) and (**) indicate a significant difference between compared groups, with *p*-values of less than 0.05 and 0.01. (*N* = 4).

The Welch’s test in Statistical Analysis of Metagenomic Profiles (STAMP) was assessed to find out the compositional differences in gut microbiota between each group. Significant reduction in abundances of *Acetivibrio alkalicellulosi*, *Blautia intestinalis*, and *Spiroplasma eriocheris* CCTCC M 207170 was observed after HFD feeding, while *Fumia xinanensis* and *Corynebacterium dentalis* had significantly increased. Surprisingly, FER supplementation could preserve the abundance of *Blautia intestinalis*, while DFER could protect against the reduction in *Acetivibrio* and *Fumia xinanensis*. In addition, both samples could facilitate the growth of *Dubosiella newyorkensis* (Figure 5).

**Figure 6:**
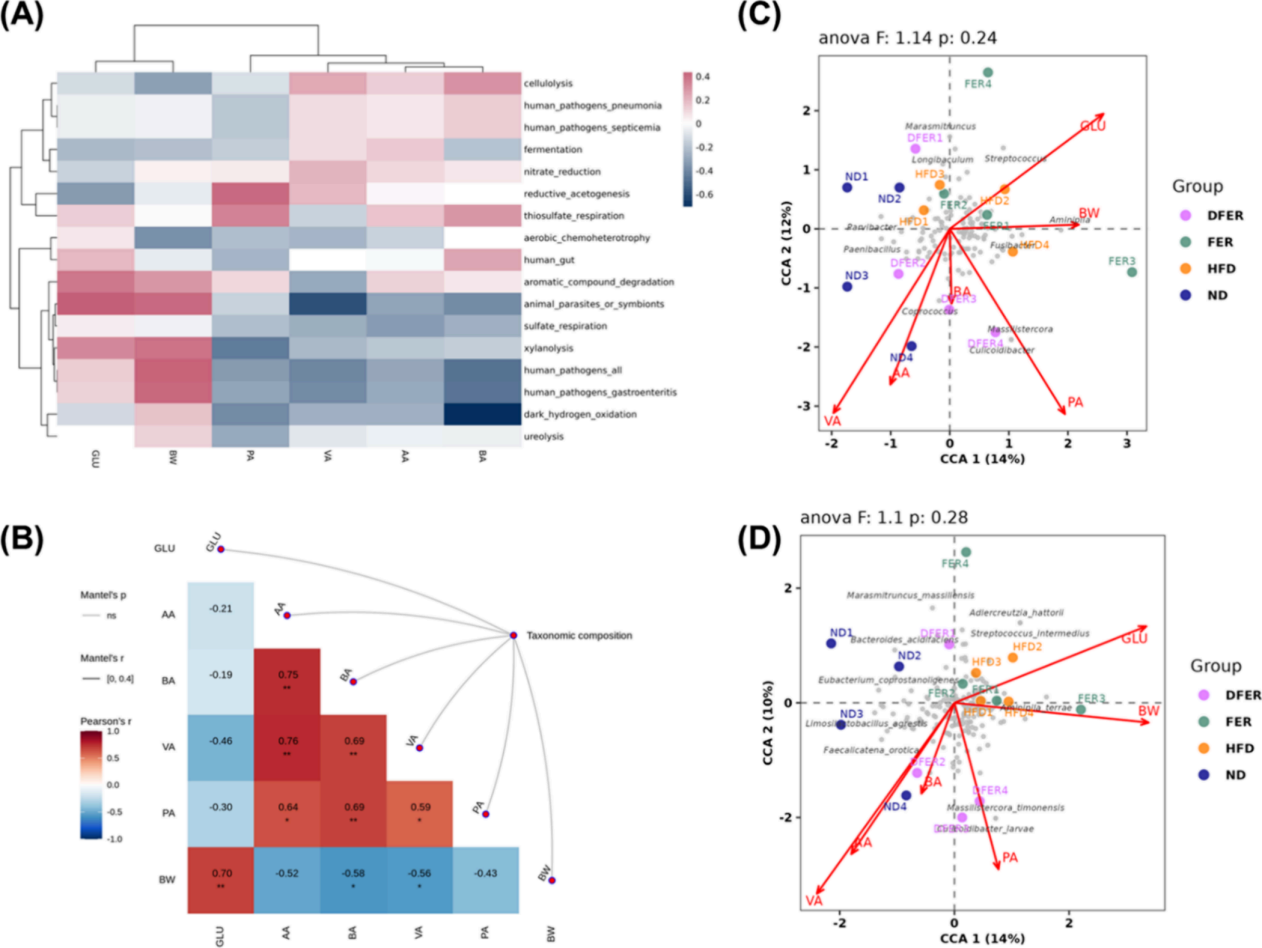
Correlation between predicted gut microbial functionality and gut microbial SCFAs. (A) Predicted correlation between gut microbial SCFAs and gut microbial functionality predicted by using the FAPROTAX database. (B) Partial Mantel tests between the SCFAs and the taxonomic compositions. The heatmap shows the pairwise correlations among selected variables: BW = body weight, GLU = fasting serum glucose, AA = fecal acetic acid, PA = fecal propionic acid, BA = fecal butyric acid, and VA = fecal valeric acid. The color represents Pearson’s correlation coefficient. Canonical correspondence analysis (CCA) plot of bacterial (C) genera and (D) species and selected factors.

According to Figure 6A, fecal acetic acid, propionic acid, butyric acid, and valeric acid levels were found to be negatively correlated to human pathogens—human pathogens that lead to gastroenteritis and animal parasites. These SCFAs were negatively correlated to body weight and blood glucose levels (Figure 6B). Some of the gut microbes were found to be positively correlated to the fecal level of SCFAs, including *Massilistercora timonensis* and Culicoidibacter, while *Streptococcus intermedius*, *Marasmitruncus massiliensis*, and *Adlercreutzia hattorii* were in negative correlation (Figure 6C,D). *Aminipilla terrae* was found to have a positive correlation to body weight gain, while *Streptococcus intermedius* showed a positive correlation to blood glucose level.

### The Preventive Effect of FER and DFER in Different Cell Models

**Figure 7:**
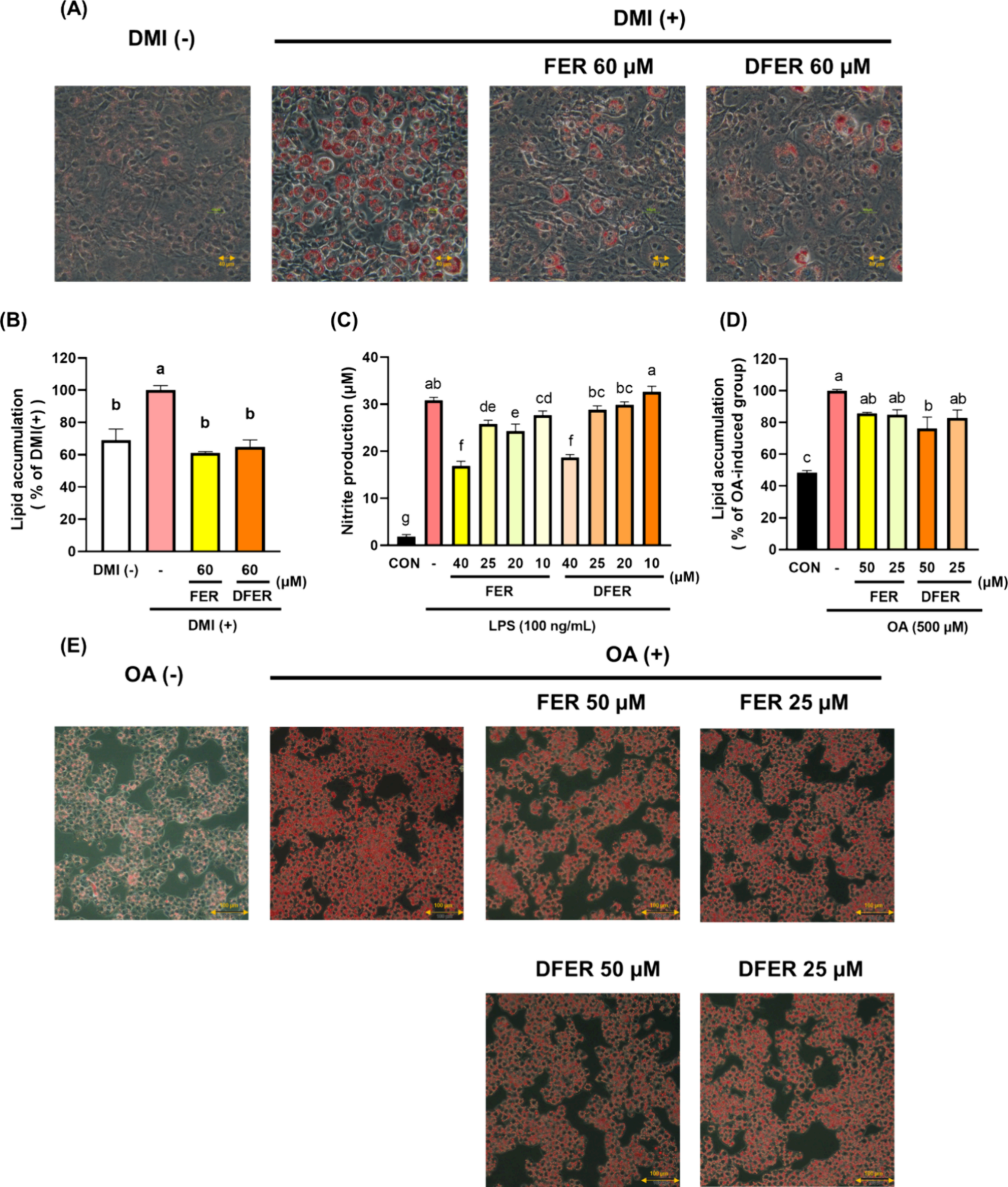
Preventive effect of FER and DFER in different cell models. (A) Oil red O-stained representative images of differentiated 3T3-L1 mature adipocytes treated with FER or DFER at 60 μM. (B) Quantification of lipid accumulation in (A). (C) NO production after murine macrophage RAW264.7 was induced by LPS for 24 h. (D) Quantification of lipid accumulation in (E). (E) Oil red O-stained representative images of oleic-induced HepG2 treated with/without FER and DFER for 24 h. Different lowercase letters indicate a significant difference among the groups, as determined by ANOVA followed by Duncan’s post hoc test.

Due to the distinct effects of FER and DFER revealed in an animal study, we employed some cell models to confirm our findings. In the induction of 3T3-L1 preadipocyte differentiation, both FER and DFER exhibited a reduction in the number of accumulated oil droplets at a concentration of 60 μM. However, there was no significant difference between FER and DFER in terms of their ability to inhibit differentiation-induced oil droplet formation (Figure 7A).

By use of oleic acid at a concentration of 500 μM in the HepG2 cell line, lipid accumulation in liver cells can be mimicked. Our results showed that both samples could consistently reduce lipid accumulation (Figure 7D). Regarding the anti-inflammatory effect, both samples could significantly reduce NO production, with no significant difference between them (Figure 7C). Our results indicate that both samples could exhibit a similar effect on obesity-related lipid accumulation or inflammatory response. Therefore, their modulatory effect on gut microbial SCFA production could be responsible for the distinct ameliorative effect observed in the animal study.

### The Preventive Effect of FER and DFER on HFD-Induced Obesity in Mice at Lower Supplementation Dosage

Due to the evident effects of FER and DFER on obesity in mice, we reduced the supplementation dosage of FER and DFER in the animal study to validate our findings (Figure S2). As anticipated, DFER still demonstrated a superior antiobesity effect in terms of the weight gained due to HFD feeding (Figure S2A), with no significant difference observed between the HFD and lower FER group (LF group). Surprisingly, both samples exhibited a positive effect on reducing weights in different adipose tissues and body fat ratios (Figure S2E–I). It was unexpected that there was no significant difference between the LF and LDF groups in terms of the adipose tissue weight. Our results suggest that both samples could still demonstrate a positive effect on inhibiting weight gain at a lower dosage, but further experiments should be conducted to confirm their efficacy.

## Discussion

In this study, we investigated the positive impact of the curcuminoid degradants FER and DFER. Our results showed that both samples could effectively prevent weight gain, hepatic lipid accumulation, and adipose tissue enlargement through dietary supplementation. Surprisingly, DFER exhibited a better effect than FER on lipid metabolism regulation and promoting thermogenesis. Moreover, FER and DFER demonstrated a distinct effect on the cecal SCFA composition. It is worth mentioning that the increased production of butyric acid in the FER supplementation group could possibly contribute to the modulation of the hepatic inflammatory response.

A previous study identified several thermal degradants produced from curcumin, including vanillin, vanillic acid, and ferulic acid, but there are some other degradation products that remained unidentified.^25^ In comparison to those identified degradants, both FER and DFER preserve the β-diketone group and olefinic linker. The only structural difference between FER and DFER is the presence of a methoxy group on the aromatic ring. In other words, DFER could be a degradation product of DMC and BDMC. According to our findings, the absence of the methoxy group might contribute to the antiobesity effect of DFER compared to FER.

A recent study comparing the antidifferentiation effect of three curcuminoids pointed out the role of the methoxy group therein.^26^ The study demonstrated a more obvious inhibitory effect of BDMC on lipid accumulation in 3T3-L1 compared to that of DMC and curcumin at the same concentration. A greater negative CDOCKER energy indicated that BDMC could have a comparatively stronger binding interaction with PPARγ, C/EBPα, and ACC, which might, in turn, reduce the transcription of lipogenesis-related protein expression.^26^ Moreover, it was also speculated that the absence of the methoxy group might facilitate the penetration of DFER across the membrane due to a smaller molecular size. A similar finding was demonstrated by Lee et al., who reported a better effect of dietary BDMC on inhibiting liver steatosis via downregulating the mRNA levels of genes related to hepatic lipid accumulation, possibly due to its stronger effect on AMPK activation.^27^ These studies could partly explain the better efficacy of DFER than of FER in reducing fat enlargement and hepatic lipid accumulation in our study (Figure 2).

On the other hand, the greater hepatic anti-inflammatory effect exhibited by FER compared to DFER can also be explained by structural differences. Sandur et al. demonstrated that curcumin had the most effective inhibitory effect on NF-κB activation among all the compared curcuminoids, while BDMC showed the lowest inhibition ability.^28^ The crucial importance of the methoxy group in the anti-inflammatory capability of curcuminoids was explained in a study in 2017. In this study, the methoxy group was substituted by several different functional groups, and the anti-inflammatory ability of curcumin was assessed in terms of nitrite production, iNOS and COX2 expressions, and activation of the NF-κB signaling pathway.^29^ This finding was further confirmed in a DSS-induced colitis mouse model, showing that the beneficial effect of curcumin was diminished when the methoxy groups were substituted. In our study, the fact that FER showed a better effect on immune response modulation is in agreement with those of previous studies, but further investigation is needed.

Another distinct result arising from dietary supplementation with FER and DFER was the SCFA content in the cecum (Figure 4D–I). Specifically, DFER exhibited a greater effect on the elevation of acetic acid and valeric acid contents (Figure 4D,H), while FER led to an incremental increase in the butyric acid level (Figure 4F). Additionally, both samples reversed the reduction of propionic acid and isobutyric acid levels induced by HFD feeding (Figure 4E,G). It has been previously described that intraperitoneal injection with acetic acid could increase thermogenesis, while oral administration of acetate at 10 mL/kg body weight could effectively activate AMPK. Moreover, a decrease in hepatic lipid accumulation and upregulation of PPARα and CPT1 were also observed.^30^ The higher level of acetic acid in the DFER group might explain the elevated level of thermogenesis (Figure 4) and the upregulation of hepatic p-AMPK, PPARα, and CPT1 (Figure 3) observed in our study. On the other hand, the higher level of butyric acid was previously reported to increase the level of cytokine IL-10 and decrease IL-17 by modulating B cell, dendritic cells, and neutrophil functions.^31^ Similarly, the hepatic levels of IL-17 and IL-10 were significantly modulated in the FER group in comparison (Figure 4J,K).

Among the changes observed in gut microbiota, the abundance of *Dubosiella newyorkensis* was sharply increased in both of the sample-supplemented groups (Figure 5). The beneficial effects of *Dubosiella newyorkensis* have been reported in some recent studies, including antiaging,^32^ modulating immune tolerance in colitis,^33^ and improving hypertension by altering bile acid metabolism.^34^ It has also been suggested as a potential probiotic to improve obesity and liver disease. Supplementation of FER and DFER in the diet could significantly facilitate the growth of *Dubosiella newyorkensis*, which might contribute to its antiobesity effect.^32^*Blautia* has been previously reported as an SCFA-producing genus that could potentially modulate gut microbiota composition.^35^ Two species members, *Blautia hansenii* and *Blautia producta*, are negatively correlated with visceral fat enlargement, possibly due to acetic acid and butyric acid production.^36^ Moreover, oral administration of *Blautia wexlerae* ameliorated obesity and type II diabetes in a previous study.^37^ Our results showed that FER supplementation could lead to a significant elevation in fecal acetic and butyric acids, which is consistent with the elevation of *Blautia* (Figure 5). However, the role and functionality of *Blautia intestinalis* should be further investigated.

*Acetivibrio* was the genus that sharply decreased after HFD feeding compared with the CON group and could be preserved by DFER feeding (Figure 5E). The abundance of *Acetivibrio* as an SCFA-producer was reported to activate the AMPK-SIRT1 pathway, contributing to the protection of dietary tryptophan against intestinal inflammation response.^38^ DFER supplementation could effectively preserve the growth of *Acetivibrio*, accompanied by significantly higher fecal acetic acid, which might assist in the ameliorative effect on obesity.

In this study, some bacteria—such as *Streptococcus intermedius*—were found to be positively correlated to increased blood glucose and body weight while negatively correlated to fecal SCFAs. It was reported that the risk of disease caused by *Streptococcus* infection was found to increase in adults with obesity and diabetes.^39^ On the other hand, *Massilistercora timonensis* has an inverse correlation to the serum cholesterol, showing its potential in lipid regulation.^40^

In summary, our study is the first to investigate and discuss the physiological function of curcuminoid degradants, FER and DFER, in HFD-induced obesity in a mouse model (Table 2). It was revealed that both samples could exhibit a lipid regulatory effect, promote thermogenesis, and have a positive impact on SCFA levels. Surprisingly, DFER showed a better overall antiobesity effect. However, it should not be neglected that FER exhibits an eye-catching effect on inflammatory responses. Additionally, the modulatory effects of FER and DFER might crucially contribute to their beneficial effects. Further studies are expected to be conducted on the efficacy of curcuminoid degradants, including toxicity and pharmacokinetic evaluations, in the future.

## Data Availability

The data that support the findings of this study are available in the Supporting Information of this article.

## Supporting Information Available

(Figure S1) LC-MS chromatograms of demethoxyferuloyacetone (DFER), feruloylacetone (FER), bisdemethoxycurcumin (BDMC), demethoxycurcumin (DMC), and curcumin (CUR) and (Figure S2) antiobesity effect of FER and DFER at lower dosage (PDF)

## Notes

The animal study was conducted following ethical guidelines and was approved by the Institutional Animal Care and Use Committee of the National Taiwan University with the approval number NTU-111-EL-00088.
