# Supplementary material for: Structural Variances in Curcumin Degradants: Impact on Obesity in Mice
Source: J Agric Food Chem. 2024 Jun 21;72(26):14786–98. doi: 10.1021/acs.jafc.4c03768 (PMC11228970; doi:10.1021/acs.jafc.4c03768)
Supplement: Supplementary file 1 — jf4c03768_si_001.pdf [file jf4c03768_si_001.pdf]

Supporting Information

Structural Variances in Curcumin Degradants: Impact on Obesity in Mice

Yen-Chun Koh[1#], Han-Wen Hsu[1#], Pin-Yu Ho[1], Kai-Yu Hsu[1], Wei-Sheng Lin[1,2], Kalyanam Nagabhushanam[3], Chi-Tang Ho[4], Min-Hsiung Pan[1,5,6*]

[1] Institute of Food Sciences and Technology, National Taiwan University, 10617 Taipei, Taiwan

[2] Department of Food Science, National Quemoy University, 89250 Quemoy County, Taiwan

[3] Sabinsa Corporation, East Windsor, 08520 New Jersey, USA

[4] Department of Food Science, Rutgers University, New Brunswick, 08901 New Jersey, USA

[5] Department of Medical Research, China Medical University Hospital, China Medical University, 40402 Taichung City, Taiwan

[6] Department of Health and Nutrition Biotechnology, Asia University, 41354 Taichung City, Taiwan

#The authors contribute equally

***Please send all correspondence to:**

Dr. Min-Hsiung Pan

Institute of Food Science and Technology,

National Taiwan University,

No. 1, Section 4, Roosevelt Road, Taipei 10617, Taiwan.

Tel. no. +886-2-33664133

Fax. no. +886-2-33661771

***E-mail: mhpan@ntu.edu.tw**

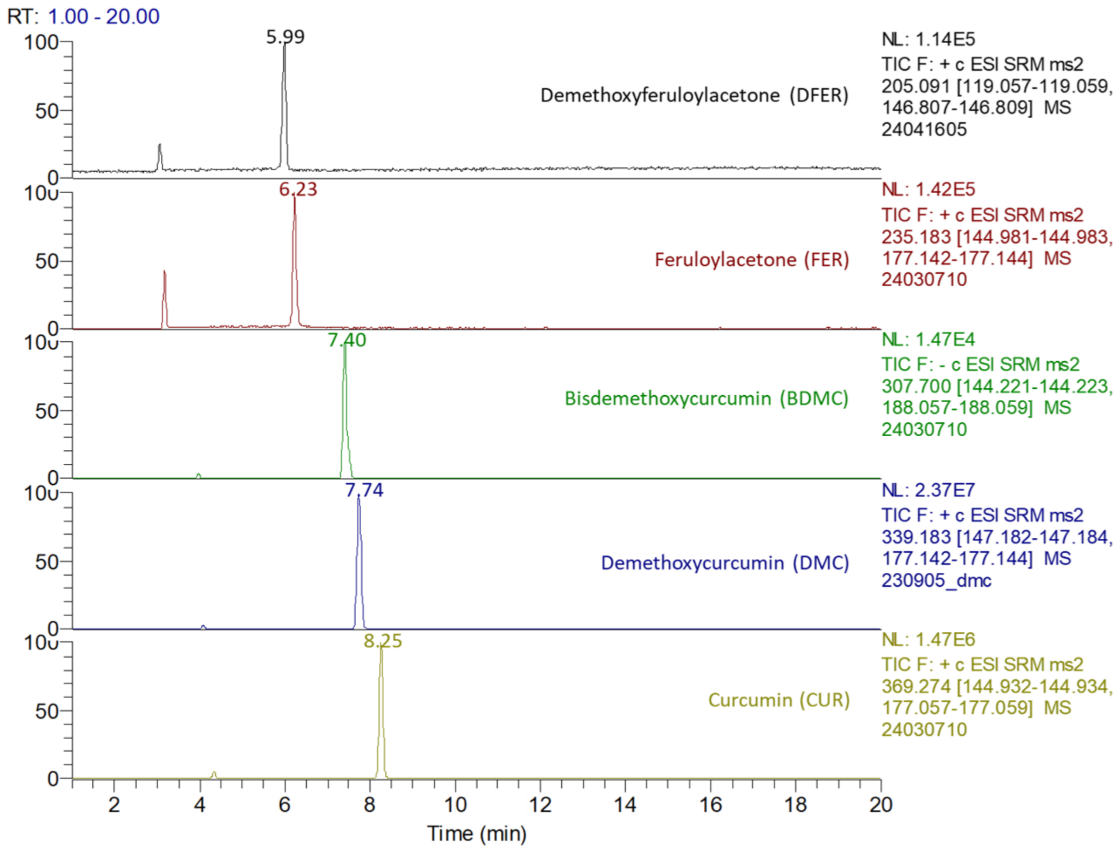

Figure S1. LC-MS chromatograms of demethoxyferuloyacetone (DFER), feruloylacetone (FER), bisdemethoxycurcumin (BDMC), demethoxycurcumin (DMC) and curcumin (CUR).

For the identification of curcumin, demethoxycurcumin, bisdemethoxycurcumin, feruloyacetone and demethoxyferuloylacetone, the protocol was as follows: using a ZORBAX Eclipse Plus C18 column (150 × 4.6 mm ID, particle size 5 μm) from Agilent Co (Santa Clara, CA, USA) and a gradient mobile phase consisting of (A) 0.1% formic acid solution and (B) acetonitrile, curcuminoids and related compounds were separated. The flow rate was set at 0.7 mL/min, and the column temperature was maintained at 25 °C. The gradient elution started with 60% A and 40% B, increased to 64% B over 10 minutes, held for 4 minutes, and then increased to 90% B over 20 minutes [1]. Detection of the various components in the samples was carried out using selected reaction monitoring (SRM) mode on a Dionex Ultimate 3000 model Open Sampler XRS UHPLC system, coupled with a TSQ Quantiva triple quadrupole tandem mass spectrometer with electrospray ionization mode from Thermo Fisher Scientific Co. (San Jose, CA, USA).

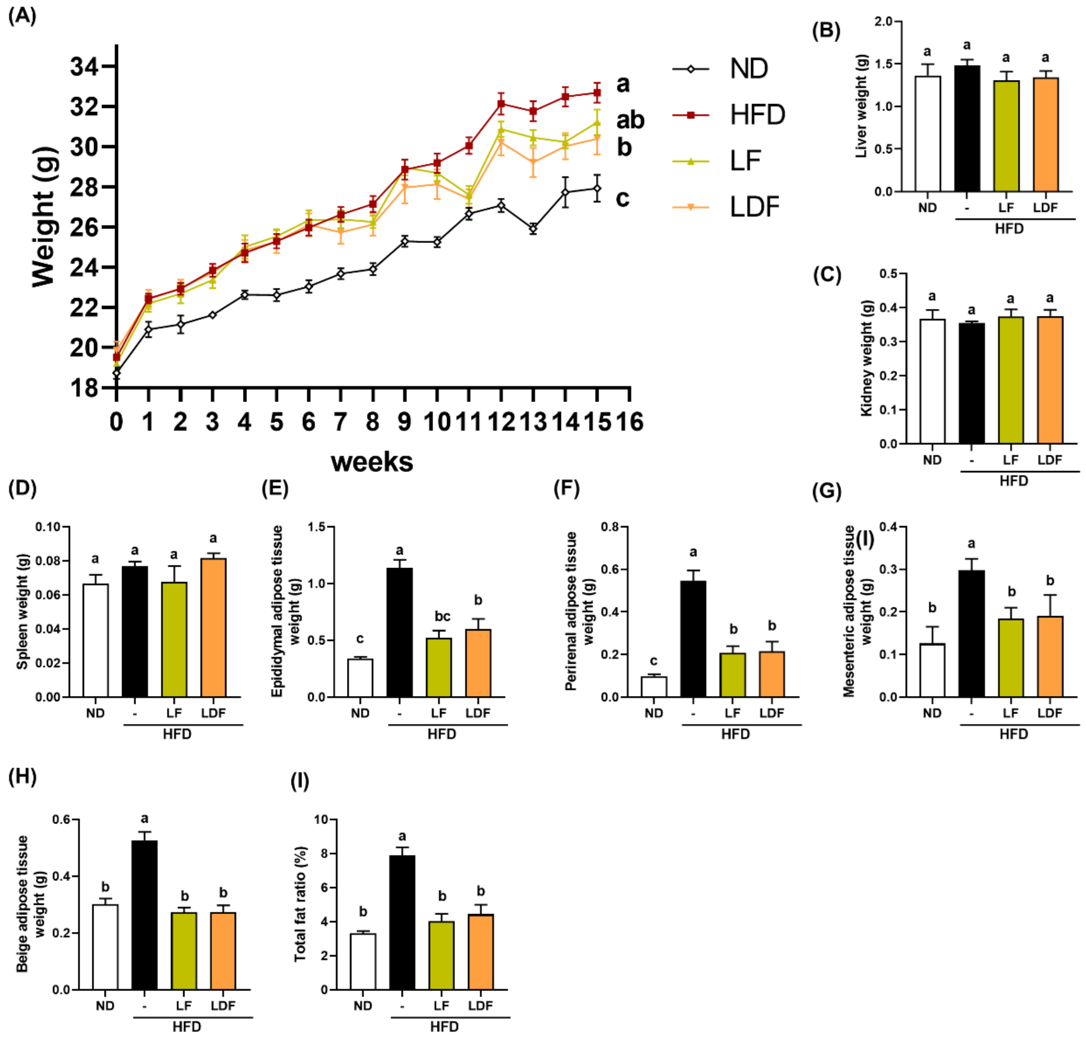

Figure S2. DFER showed a greater effect on preventing weight gain in HFD-induced obesity in mice at lower supplementation dosage

(A) The body weight change of the HFD-fed mice supplemented with 0.1% FER or 0.1% DFER in diet for 15 weeks, the average weight of (B) liver, (D) kidney, and (E) spleen, (F) epididymal WAT, (G) perirenal WAT, (H) mesenteric WAT, (I) beige adipose tissue, and the total fat ratio (%). All the data are presented as mean ± S.E, N=7. Different lowercase letters indicate a significant difference among the groups, as determined by ANOVA followed by Duncan's post hoc test.

1. Lu, P. S.; Inbaraj, B. S.; Chen, B. H., Determination of oral bioavailability of curcuminoid dispersions and nanoemulsions prepared from Curcuma longa Linnaeus. *Journal of the Science of Food and Agriculture* **2018,** *98* (1), 51-63.
